# Spectrum of malignancies among the population of adults living with HIV infection in China: A nationwide follow-up study, 2008–2011

**DOI:** 10.1371/journal.pone.0219766

**Published:** 2019-07-25

**Authors:** Weiming Zhu, Yurong Mao, Houlin Tang, Jennifer M. McGoogan, Zuo-Feng Zhang, Roger Detels, Na He, Zunyou Wu

**Affiliations:** 1 National Center for AIDS/STD Control and Prevention, Chinese Center for Disease Control and Prevention, Beijing, China; 2 Department of Epidemiology, Fielding School of Public Health, University of California–Los Angeles, Los Angeles, California, United States of America; 3 Department of Epidemiology, School of Public Health, Fudan University, Shanghai, China; Centre de Recherche en Cancerologie de Lyon, FRANCE

## Abstract

**Background:**

Although increasingly studied in high-income countries, there is a paucity of data from the Chinese population on the patterns of cancer among people living with HIV (PLHIV).

**Methods:**

We conducted a nationwide follow-up study using routinely collected data for adult PLHIV diagnosed on or before 31 December 2011 and alive and in care as of 1 January 2008. Participants were observed from 1 January 2008 (study start) to 30 June 2012 (study end). Main outcome measures were gender-stratified age-standardized incidence rates for China (ASIRC) and standardized incidence ratios (SIR) for all malignancy types/sites observed.

**Results:**

Among 399,451 subjects, a majority was aged 30–44 years (49.3%), male (69.8%), and Han Chinese (67.9%). A total of 3,819 reports of cancer were identified. Overall, ASIRC was 776.4 per 100,000 for males and 486.5 per 100,000 for females. Malignancy sites/types with highest ASIRC among males were lung (226.0 per 100,000), liver (145.7 per 100,000), and lymphoma (63.1 per 100,000), and among females were lung (66.8 per 100,000), lymphoma (48.0 per 100,000), stomach (47.8 per 100,000), and cervix (47.6 per 100,000). Overall SIR for males was 3.4 and for females was 2.6. Highest SIR was observed for Kaposi sarcoma (2,639.8 for males, 1,593.5 for females) and lymphoma (13.9 for males, 16.0 for females).

**Conclusions:**

These results provide evidence of substantial AIDS-defining and non-AIDS-defining cancer burden among adult Chinese PLHIV between 2008 and 2011. Although further study is warranted, China should take action to improve cancer screening, diagnosis, and treatment for this vulnerable population.

## Introduction

Substantial evidence supports a strong link between HIV infection and increased incidence of a broad spectrum of malignancies. In the early 1980s, the US Centers for Disease Control and Prevention (CDC) identified Kaposi sarcoma (KS), non-Hodgkin lymphoma (NHL), and invasive cervical cancer (ICC) as AIDS-defining cancers (ADCs). Diagnosis of any one ADC marks progression to AIDS [1]. Prior to the introduction of antiretroviral therapy (ART) in the mid-1990s, incidence of ADCs among people living with HIV (PLHIV) was substantially elevated over that of the general population. However, since the widespread scale up of ART, cancer types/sites observed among PLHIV appears to have changed—incidence of KS and NHL has fallen dramatically, while incidence of non-AIDS-defining cancers (NADCs) has risen [2–7].

In general, NADCs can be categorized by their infectious versus non-infectious etiologies. A range of anogenital cancers and head and neck cancers, as well as some non-melanoma skin cancers are associated with the oncogenic subtypes of Human Papillomavirus (HPV). Epstein-Barr Virus (EBV), and in some cases, Human Herpesvirus 8 (HHV-8, the virus that causes KS), is involved in many of the nine different subtypes of lymphomas, while Hepatitis B Virus (HBV) and Hepatitis C Virus (HCV) are associated with liver cancer [3,8]. Not surprisingly, PLHIV are known to be more susceptible to infection with these oncogenic viruses, and immunodeficiency may also interact with the oncogenesis. However, PLHIV are also known to have higher rates of exposure to cigarette smoke and alcohol, two of the top non-infectious carcinogens, which may also partially explain the higher rates of not only lung and liver cancers among PLHIV, but also cancers of the head and neck and cervix. Although the rise in NADCs may simply be a consequence PLHIV having increased longevity in the ART era, there is evidence suggesting that there are other factors at play. Compared to the general population, PLHIV are more often diagnosed at earlier ages with more aggressive cancers that have already progressed to later stages [3,9].

Most cancer research has been performed among PLHIV of European or African heritage [5–7,10–26]. Previous studies in Chinese populations (two hospital-based studies in Mainland China, with 3,554 and 1,946 patients, respectively, and one registry-based study with 15,269 patients in Taiwan) reported that the risk of KS, NHL, cervical, liver, and anal cancers were elevated among PLHIV compared to the general population [27–29]. However, the spectrum of malignancies observed among China’s rapidly growing population of PLHIV has not yet been systematically described. Furthermore, the cancer spectrum in the general Chinese population differs from many developed countries. For instance, China has a higher incidence of liver, esophagus, nasopharynx, stomach, and lung cancers [30]. It is also not clear whether such differences in cancer spectra exist between PLHIV in China, and PLHIV in other countries.

Thus, we conducted a nationwide follow-up study using data from China’s National HIV/AIDS Surveillance Program and National Free ART Program (NFATP) to retrospectively investigate the spectrum of malignancies among adult PLHIV in China.

## Materials and methods

### Study design

We conducted a nationwide follow-up study to examine the spectrum of malignancies among Chinese adult PLHIV. To do this, we used routinely collected data for PLHIV who had been diagnosed with HIV infection on or before 31 December 2011 and were still alive and in care as of 1 January 2008. All participants contributed observed time from 1 January 2008 (i.e., the study start date) to 30 June 2012 (i.e., the study end date) and cancer cases were identified during this follow-up time.

### Setting and data source

The data used for this study were collected during the normal operation of China’s HIV/AIDS Surveillance System and NFATP. All data related to China’s HIV response efforts are collected and maintained in a real-time, web-based repository called the HIV/AIDS Comprehensive Response Information Management System (CRIMS), which has been described elsewhere [31]. In brief, CRIMS contains records for all individuals diagnosed with HIV in China, and these records include contact and demographic information, testing and baseline clinical information, dates and details of all follow-up visits, and ART regimens and related assessments, as well as other health-related information (co-infections, co-morbidities) and dates and causes of death. After a diagnosis of HIV infection, but prior to the initiation of ART, PLWH are followed up once every 6 months. After ART initiation, patients are followed up 4 times in the first 3 months, and once every 3 months thereafter.

### Eligibility criteria

Study inclusion criteria were: 1) being ≥15 years of age, and 2) having been diagnosed with HIV on or before 31 December 2011. All patient records in CRIMS meeting these study inclusion criteria were extracted and then screened against exclusion criteria. Study exclusion criteria were: 1) having died or been lost to follow-up prior to the start of the study on 1 January 2008, or 2) not having attended at least one follow-up visit prior to 30 June 2012, the date all data were extracted. All remaining patients were included in the analysis.

### Case identification

Cancer cases were identified using reports of AIDS-related complications or causes of death at each follow-up visit. We categorized cancer cases based on the International Classification of Disease for Oncology, 3rd Edition (ICD-O-3) [32]. However, because approximately half of lymphoma records were not specified as to their type, we combined all cases of NHL, Hodgkin lymphoma (HL), and all other lymphomas together into a single category. This group was treated as a single cancer type in all analyses.

### Observed time

The start of observed time was defined as either the study start date, 1 January 2008 (for those already diagnosed with HIV), or the date of HIV diagnosis, whichever was later. The inferred date of cancer incidence was defined as the median date between the date of cancer diagnosis and the date of the previous follow-up visit. Participants with inferred date of cancer incidence after the end of the study, 31 December 2011, were treated as cancer-free during the study period. For cancer-free individuals, the end of follow-up was defined as either 1) the date of last follow-up for those who were lost to follow-up, 2) the date of death, or 3) the end of the study for those who were still living. Observed time was calculated as the difference between first and last observation, expressed in person-years (PY).

### Statistical analysis

Characteristics of participants were presented as number and percent. Sex-specific cancer incidences were calculated for each malignancy site/type, and age-standardized incidence rates (ASIR) and standardized incidence ratios (SIR), along with their 95% confidence intervals (CIs) were calculated. Direct standardization was used to calculate ASIR. Two different standard populations were used: ASIR-China (ASIRC) was calculated using China’s 2010 census data [33], and ASIR-world (ASIRW) was calculated using the World Health Organization (WHO) standard world population for 2002 to 2025 [34]. ASIRC and ASIRW were expressed per 100,000 PY. SIR was obtained by dividing the numbers of observed cases (numerator) by the expected numbers of cases (denominator). Expected cancer frequencies were determined using the observed sex- and 5-year, age-specific incidence rates from China’s National Cancer Registry in 2008 [35]. All CIs presented were calculated assuming a Poisson distribution. SAS software version 9.3 (SAS Institute Inc., USA) was used for all analyses.

### Ethical considerations

This study was approved by the Institutional Review Board of the National Center for AIDS/STD Control and Prevention, China CDC, and by the Institutional Review Board of the University of California, Los Angeles. No informed consent was sought since all individuals diagnosed with HIV in China sign informed consent at the time of their initial entry into CRIMS, which includes the future use of their data for epidemiological study. All records, once extracted, were de-identified to ensure confidentiality.

## Results

Records of a total of 444,712 patients who met inclusion criteria were extracted. A total of 45,261 (10.2%) were excluded and therefore, 399,451 (89.8%) patients were included in the analysis and contributed a total of 813,238.9 PY of observed time.

As shown in Table 1, a majority were 30–44 years of age (49.3%), male (69.8%), and Han Chinese (67.9%), and most had a junior high school-level education or less (74.3%). The most common HIV transmission route reported by participants was heterosexual contact (43.5%), followed by injecting drug use (27.3%), and blood product receipt or donation (10.2%). At the time of diagnosis, 62.0% of participants had not yet progressed to AIDS, while 38% had, and baseline CD4 counts were low—25.6% had ≥350 cells/mm^3^, 22.1% had 200–399 cells/mm^3^, and 27.2% had <200 cells/mm^3^.

**Table 1:** Characteristics of participants—Adults diagnosed with HIV infection prior to January 1, 2012, yet still alive and being followed prior to January 1, 2008.

Baseline Characteristics	Participants N (%)	Observed Time PY (%)
**Overall**	**399,451 (100)**	**813,238.9 (100)**
**Age at HIV diagnosis, years**^**a**^		
15–29	116,082 (29.1)	213,789.8 (26.3)
30–44	196,848 (49.3)	440,565.2 (54.2)
45–59	60,917 (15.3)	124,932.1 (15.4)
≥60	25,604 (6.4)	33,951.8 (4.2)
**Gender**		
Male	278,908 (69.8)	550,228.6 (67.7)
Female	120,543 (30.2)	263,010.3 (32.3)
**Ethnicity**		
Han Chinese	271,375 (67.9)	557,772.6 (68.6)
Other	128,076 (32.1)	255,466.4 (31.4)
**Education Level**		
Illiterate	37,577 (9.4)	75,273.2 (9.3)
Primary school	112,932 (28.3)	232,735.9 (28.6)
Junior high school	146,270 (36.6)	303,861.8 (37.4)
High school	41,180 (10.3)	74,439.9 (9.2)
≥College	22,881 (5.7)	36,115.5 (4.4)
Missing	38,611 (9.7)	90,812.7 (11.2)
**HIV Transmission Route**		
Heterosexual contact	173,853 (43.5)	290,022.6 (35.7)
Injecting drug use	108,911 (27.3)	259,168.0 (31.9)
Blood product receipt or donation	40,860 (10.2)	128,043.6 (15.7)
Homosexual contact	29,169 (7.3)	40,022.4 (4.9)
Sexual contact and injecting drug use	4,900 (1.2)	10,179.8 (1.3)
Other/Unknown/Missing	41,758 (10.5)	85,802.6 (10.6)
**Disease Stage at Diagnosis**		
HIV-infection	247,625 (62.0)	445,270.7 (54.8)
AIDS	151,826 (38.0)	367,968.3 (45.2)
**CD4 Count (cells/mm**^**3**^**)**		
<200	108,585 (27.2)	210,185.4 (25.8)
200–349	88,439 (22.1)	196,043.6 (24.1)
≥350	102,305 (25.6)	250,856.9 (30.8)
Missing	100,122 (25.1)	156,153.1 (19.2)

PY: person-years

^a^According to the National Bureau of Statistics of China [33], the age distribution of the general population in 2010 was: 16.6% ≤14 years of age, 74.5% 15–64 years of age, and 8.9% ≥65 years of age

As shown in Table 2, 3,819 cancer cases were identified—2,808 among males, and 1,011 among females. The 5 most prevalent malignancies among males were lung cancer (n = 713), liver cancer (n = 539), lymphoma (n = 299), brain and central nervous system (CNS) cancers (n = 216), and stomach cancer (n = 137), and among females were lung cancer (n = 140), cervical cancer (n = 128), lymphoma (n = 117), brain and CNS cancers (n = 105), and liver cancer (n = 84).

**Table 2:** Gender-stratified frequencies of malignancies and age-standardized incidence rates weighted for China (ASIRC) and for the world (ASIRW), 2008 to 2011.

Malignancy Site/Type (ICD-O-3 Code)	Males, per 100,000			Females, per 100,000		
	N	ASIRC^a^ (CI)	ASIRW^b^ (CI)	N	ASIRC^a^ (CI)	ASIRW^b^ (CI)
**All Cancers**	**2,808**	**776.4 (742.1–810.6)**	**881.7 (839.6–923.7)**	**1,011**	**486.5 (436.4–536.6)**	**508.9 (454.0–563.7)**
All (Excluding Ill-defined/Unspecified)	2,524	691.8 (659.7–724.0)	782.7 (743.3–822.0)	898	427.4 (380.5–474.2)	446.0 (394.7–497.3)
Ill-defined/Unspecified	284	84.6 (72.8–96.3)	99.0 (84.2–113.9)	113	59.2 (41.5–76.8)	62.8 (43.5–82.1)
**AIDS-Defining Cancers (all lymphomas**^**c**^**)**						
Kaposi sarcoma (9140)	132	23.9 (18.9–28.8)	23.9 (18.5–29.2)	39	14.2 (9.0–19.4)	13.9 (8.5–19.2)
Lymphoma^c^ (959, 965)	299	63.1 (54.6–71.7)	65.8 (56.3–75.4)	117	48.0 (32.7–63.2)	48.8 (32.3–65.2)
Cervical (C53)	-	-	-	128	47.6 (38.3–57.0)	48.0 (38.3–57.8)
**Non-AIDS-Defining Cancers (no lymphomas**^**c**^**)**						
Oral cavity (C00–C09)	13	5.3 (2.1–8.4)	6.6 (2.3–10.8)	4	1.9 (0.0–3.9)	2.0 (0.0–4.2)
Nasopharynx (C11)	38	11.0 (7.1–15.0)	12.0 (7.6–16.3)	9	3.8 (1.1–6.6)	4.3 (1.1–7.4)
Other head and neck (C12–C14, C30–C32)	47	13.8 (9.1–18.6)	15.9 (10.0–21.7)	17	7.6 (3.3–11.8)	8.0 (3.4–12.7)
Esophagus (C15)	78	25.3 (19.0–31.7)	29.0 (21.4–36.7)	19	14.8 (1.8–27.9)	16.1 (1.7–30.4)
Stomach (C16)	137	44.1 (35.6–52.6)	52.6 (41.6–63.5)	60	47.8 (24.2–71.4)	51.4 (25.5–77.3)
Colon and rectum (C18–20)	121	34.4 (27.3–41.5)	39.2 (30.5–47.9)	32	22.0 (11.0–33.0)	23.1 (11.3–34.8)
Anus (C21)	2	0.2 (0.0–0.5)	0.2 (0.0–0.4)	0	-	-
Liver (C22)	539	145.7 (131.0–160.3)	160.8 (143.6–178.0)	84	39.2 (27.4–51.0)	41.4 (28.7–54.2)
Other digestive system (C23, C24, C26)	6	1.8 (0.2–3.4)	2.6 (0.0–5.3)	1	0.7 (0.0–2.2)	0.9 (0.0–2.6)
Pancreas (C25)	41	12.0 (7.8–16.3)	14.2 (8.8–19.5)	2	5.0 (0.0–14.3)	6.4 (0.0–18.6)
Lung (C34)	713	226.0 (206.7–245.4)	266.3 (241.8–290.8)	140	66.8 (52.5–81.1)	71.3 (55.7–86.9)
Mediastinum and pleura (C38)	4	0.7 (0.0–1.5)	0.7 (0.0–1.5)	2	0.5 (0.0–1.3)	0.5 (0.0–1.1)
Bone (C40)	11	4.2 (1.3–7.0)	5.3 (1.4–9.2)	2	0.9 (0.0–2.1)	1.0 (0.0–2.5)
Skin (C44)	17	4.6 (2.1–7.2)	5.0 (2.2–7.9)	9	3.2 (1.0–5.3)	3.2 (1.0–5.5)
Soft tissue (C47–C49)	5	1.1 (0.0–2.3)	1.1 (0.0–2.5)	7	2.1 (0.5–3.7)	2.1 (0.5–3.7)
Breast (C50)	2	0.3 (0.0–0.8)	0.3 (0.0–0.8)	31	13.6 (7.0–20.2)	14.1 (7.1–21.2)
Vagina and vulva (C51, C52)	-	-	-	2	0.7 (0.0–1.7)	0.7 (0.0–1.8)
Corpus uteri (C54)	-	-	-	48	24.9 (10.8–39.0)	26.2 (10.9–41.6)
Ovary and other female genital tissue (C55–C58)	-	-	-	5	1.7 (0.2–3.1)	1.7 (0.1–3.4)
Penis (C60)	6	2.1 (0.2–3.9)	2.4 (0.3–4.5)	-	-	-
Prostate (C61)	9	4.3 (1.3–7.3)	6.1 (1.7–10.5)	-	-	-
Other male genital (C62, C63)	5	1.1 (0.0–2.5)	1.2 (0.0–2.6)	-	-	-
Kidney (C64)	10	2.5 (0.5–4.5)	2.9 (0.4–5.3)	2	0.6 (0.0–1.5)	0.6 (0.0–1.5)
Other urinary system (C65–C68)	3	0.8 (0.0–1.8)	0.9 (0.0–2.0)	0	-	-
Bladder (C67)	9	3.8 (1.1–6.6)	5.6 (1.2–9.9)	1	0.7 (0.0–2.2)	0.9 (0.0–2.6)
Eye (C69)	4	1.1 (0.0–2.4)	1.1 (0.0–2.6)	3	1.1 (0.0–2.5)	1.2 (0.0–2.6)
Brain and CNS (C70, C71)	216	45.5 (38.2–52.8)	47.4 (39.2–55.6)	105	43.0 (28.5–57.6)	43.0 (27.3–58.7)
Thyroid (C73)	2	0.4 (0.0–1.0)	0.4 (0.0–1.0)	0	-	-
Multiple myeloma (C90)	4	2.0 (0.0–4.0)	2.3 (0.0–4.6)	1	0.4 (0.0–1.3)	0.5 (0.0–1.6)
Melanoma (872)	1	0.3 (0.0–0.9)	0.4 (0.0–1.1)	0	-	-
Leukemia (981)	50	10.3 (6.8–13.8)	10.7 (6.7–14.6)	28	14.4 (6.1–22.6)	14.6 (5.9–23.4)

ICD-O-3: International Classification of Disease for Oncology [32], 3rd Edition, ASIRC: age-standardized incidence rate for China, ASIRW: age-standardized incidence rate for the world, CI: 95% confidence interval, CNS: central nervous system

^a^ASIRC is per 100,000, weighted by Chinese population in 2010 according to national census data [33], and CI calculated based on an assumed Poisson distribution

^b^ASIRW is per 100,000, weighted by the World Health Organization’s world standard population data for 2002 to 2025 [34], and CI calculated based on an assumed Poisson distribution

^c^Because approximately half of lymphoma records did not specify Hodgkin or Non-Hodgkin lymphoma, combined observed and expected lymphoma figures were calculated

Table 2 also presents sex-specific ASIRC and ASIRW results overall and by malignancy site/type. Overall ASIRC was 776.4 per 100,000 (742.1–810.6) for males and 486.5 per 100,000 (436.4–536.6) for females. Excluding cancer cases with ill-defined or unspecified primary sites/types, overall ASIRC was 691.8 per 100,000 (659.7–724.0) for males and 427.4 per 100,000 (380.5–474.2) for females. Overall ASIRW was 881.7 per 100,000 (839.6–923.7) for males and 508.9 per 100,000 (454.0–563.7) for females, higher compared to ASIRC. ASIRW was also higher than ASIRC for both males and females when malignancies that were ill-defined/unspecified were excluded.

ASIRW values were similar or greater than ASIRC values for all specific malignancy sites/types for both males and females. Lung cancer had the highest ASIRC among males at 226.0 per 100,000 (206.7–245.4) followed by liver cancer at 145.7 per 100,000 (131.0–160.3) and lymphoma at 63.1 per 100,000 (54.6–71.7). Lung cancer also had the highest ASIRC among females at 66.8 per 100,000 (52.5–81.1) followed by lymphoma at 48.0 per 100,000 (32.7–63.2), stomach cancer at 47.8 per 100,000 (42.2–71.4), and cervical cancer at 47.6 per 100,000 (38.3–57.0).

Table 3 displays results of sex-specific SIR calculations overall and by malignancy site/type. Overall SIR was 3.4 (3.3–3.5) for males and 2.6 (2.4–2.7) for females. After excluding ill-defined or unspecified malignancies, SIR for males was 3.1 (3.0–3.2) and for females was 2.3 (2.2–2.5). Highest SIR was observed for KS at 2,639.8 (2,208.7–3,130.5) among males, and 1,593.5 (1,133.0–2,178.4) among females, and lymphomas (all types) was 13.9 (12.3–15.5) among males and 16.0 (13.2–19.1) among females. Among males, high SIR was observed for cancers of the eye (10.3 [2.8–26.4]), brain and CNS (8.2 [7.1–9.3]), lung (4.8 [4.4–5.1]), and liver (3.9 [3.6–4.2]). Among females, high SIR was found for other head and neck cancers (16.0 [9.3–25.6]), and cancers of the eye (9.6 [1.9–28.1]), brain and CNS (8.8 [7.2–10.7]), lung (4.2 [3.5–5.0]), and liver (4.0 [1.8–8.0]). ICC had an SIR of 3.8 (3.2–4.6).

**Table 3:** Gender-stratified frequencies of malignancies and standard incidence ratios (SIR) for China, 2008 to 2011.

Malignancy Site/Type (ICD-O-3 Code)	Males			Females		
	Observed	Expected^a^	SIR^a^ (CI)	Observed	Expected^a^	SIR^a^ (CI)
**All Cancers**	**2,808**	**832.2**	**3.4 (3.3–3.5)**	**1,011**	**392.6**	**2.6 (2.4–2.7)**
All (Excluding Ill-defined/Unspecified)	2,524	809.9	3.1 (3.0–3.2)	898	382.2	2.3 (2.2–2.5)
Ill–defined/Unspecified	284	22.3	12.8 (11.3–14.3)	113	10.4	10.9 (9.0–13.1)
**AIDS-Defining Cancers (all lymphomas**^**b**^**)**	**431**	**21.6**	**19.9 (18.1–21.9)**	**284**	**40.7**	**7.0 (6.2–7.8)**
Kaposi sarcoma (9140)	132	0.05	2,639.8 (2,208.7–3,130.5)	39	0.02	1,593.5 (1,133.0–2,178.4)
Lymphoma^**b**^ (959, 965)	299	21.6	13.9 (12.3–15.5)	117	7.3	16.0 (13.2–19.1)
Cervical (C53)	-	-	-	128	33.4	3.8 (3.2–4.6)
**Non-AIDS-Defining Cancers (no lymphomas**^**b**^**)**	**2,377**	**810.6**	**2.9 (2.8–3.1)**	**727**	**351.9**	**2.1 (1.9–2.2)**
Oral cavity (C00–C09)	13	9.9	1.3 (0.7–2.2)	4	3.3	1.2 (0.3–3.1)
Nasopharynx (C11)	38	22.9	1.7 (1.2–2.3)	9	5.2	1.7 (0.8–3.3)
Other head and neck (C12–C14, C30–C32)	47	13.6	3.4 (2.5–4.6)	17	1.1	16.0 (9.3–25.6)
Esophagus (C15)	78	62.5	1.2 (1.0–1.6)	19	10.9	1.7 (1.0–2.7)
Stomach (C16)	137	116.1	1.2 (1.0–1.4)	60	26.7	2.2 (1.7–2.9)
Colon and rectum (C18–C20)	121	81.5	1.5 (1.2–1.8)	32	29.5	1.1 (0.7–1.5)
Anus (C21)	2	0.7	2.9 (0.3–10.5)	0	0.2	0.0 (0.0–19.7)
Liver (C22)	539	138.3	3.9 (3.6–4.2)	84	16.1	5.2 (4.2–6.5)
Other digestive system (C23, C24, C26)	6	12.1	0.5 (0.2–1.1)	1	5.3	0.2 (0.0–1.0)
Pancreas (C25)	41	19.7	2.1 (1.5–2.8)	2	6.1	0.3 (0.0–1.2)
Lung (C34)	713	150.1	4.8 (4.4–5.1)	140	33.2	4.2 (3.5–5.0)
Mediastinum and pleura (C38)	4	4.9	0.8 (0.2–2.1)	2	1.3	1.5 (0.2–5.5)
Bone (C40)	11	7.0	1.6 (0.8–2.8)	2	2.5	0.8 (0.1–2.8)
Skin (C44)	17	7.5	2.3 (1.3–3.6)	9	2.2	4.0 (1.8–7.7)
Soft tissue (C47–C49)	5	4.5	1.1 (0.4–2.6)	7	1.8	3.9 (1.5–8.0)
Breast (C50)	2	1.2	1.6 (0.2–5.8)	31	98.8	0.3 (0.2–0.4)
Vagina and vulva (C51, C52)	-	-	-	2	0.9	2.2 (0.2–8.0)
Corpus uteri (C54)	-	-	-	48	17.4	2.8 (2.0–3.7)
Ovary and other female genital tissue (C55–C58)	-	-	-	5	18.0	0.3 (0.1–0.6)
Penis (C60)	6	2.2	2.8 (1.0–6.1)	-	-	-
Prostate (C61)	9	14.8	0.6 (0.3–1.2)	-	-	-
Other male genital tissue (C62, C63)	5	5.0	1.0 (0.3–2.4)	-	-	-
Kidney (C64)	10	20.6	0.5 (0.2–0.9)	2	5.2	0.4 (0.0–1.4)
Other urinary system (C65–C68)	3	2.7	1.1 (0.2–3.3)	0	1.0	0.0 (0.0–3.5)
Bladder (C67)	9	24.0	0.4 (0.2–0.7)	1	2.9	0.4 (0.0–1.9)
Eye (C69)	4	0.4	10.3 (2.8–26.4)	3	0.3	9.6 (1.9–28.1)
Brain and CNS (C70, C71)	216	26.4	8.2 (7.1–9.3)	105	11.9	8.8 (7.2–10.7)
Thyroid (C73)	2	17.3	0.1 (0.0–0.4)	0	28.8	0.0 (0.0–0.1)
Multiple myeloma (C90)	4	3.8	1.1 (0.3–2.7)	1	1.3	0.8 (0.0–4.3)
Melanoma (872)	1	1.6	0.6 (0.0–3.6)	0	0.7	0.0 (0.0–5.2)
Leukemia (981)	50	17.2	2.9 (2.2–3.8)	28	8.8	3.2 (2.1–4.6)

ICD-O-3: International Classification of Disease for Oncology [32], 3rd Edition, SIR: standard incidence ratio, CI: 95% confidence interval, CNS: central nervous system

^a^SIR is calculated using Chinese National Cancer Registry data for 2008 (Expected) [35], and CI calculated based on an assumed Poisson distribution

^b^Because approximately half of lymphoma records did not specify Hodgkin or Non-Hodgkin lymphoma, combined observed and expected lymphoma figures were calculated

## Discussion

We observed higher incidence of cancer among Chinese adults with HIV, compared to the general Chinese population. As expected, both the incidence of ADC and NADC were higher. These findings are supported by three previous studies in ethnic Chinese populations: Zhang et al. (Hubei Province, 2004–2008) [27], Yang et al. (Beijing Municipality, 2008–2013) [28], and Chen et al. (Taiwan, 1998–2009) [29]. However, the finding of many NADCs at higher incidence rates than ADCs was somewhat of a surprise, given that ART coverage in China during the study period was <50% [36]. Further comparison of the cancer spectrum between PLHIV in different populations indicates that both infectious and non-infectious etiologies may play important roles in oncogenesis in the HIV-infected population in China.

### HHV-8 Infection

KS is well-known to be caused by HHV-8, and PLHIV are commonly infected with HHV-8. In the pre-ART era, KS incidence was observed to be up to 2,000-fold greater among PLHIV, and the more severe the immunodeficiency, the higher the likelihood of KS occurrence [8]. In our study, 171 cases of KS were observed, which represented only 4.5% of all cancer cases observed. Not surprisingly, KS SIR was high—2,639.8 among males and 1,593.5 among females—as KS in the absence of HIV is very rare in Mainland China. However, the ASIRC of KS (23.9 per 100,000 for males and 14.2 per 100,000 for females) was lower than in the Taiwan study [29], and in many previous studies in the United States, Europe and sub-Saharan Africa [5–7,10–26,37]. The low prevalence of HHV-8 infection in Mainland China may be one cause of lower KS incidence rate [38,39]. Previous studies have shown that the incidence rate and SIR of KS has dropped dramatically since the introduction of ART [5,12,18,21], and we expect a similar trend to develop in China, since ART coverage is still expanding under the NFATP as China strives to meet the Joint United Nations Programme on HIV/AIDS (UNAIDS) 90-90-90 Targets [40,41].

### EBV Infection

EBV infection is known to be associated with several forms of lymphomas, especially NHL and HL, and increased incidence of NHL has been documented among those with more serious immunodeficiency. China’s general population is known to have a high prevalence of EBV infection—one recent study found EBV prevalence among Chinese children to be 50% by age 3 and 90% by age 8 [42]. Both Zhang et al. and Chen et al. found that NHL was the most commonly observed malignancy [27,29], and Chen et al. found NHL incidence of 329 per 100,000 (SIR 23.7) for males and 256 per 100,000 (SIR 22.4) for females, whereas incidence of HL was 18 per 100,000 (SIR 9.4) for males and 14 per 100,000 (SIR 7.8) for females [29]. However, limitations in the data we collected meant we were only able to report incidence for all lymphomas as a group—SIR of 13.9 for males, 16.0 for females, and ASIRC of 63.1 per 100,000 among males and 48.0 per 100,000 among females, consistent with previous studies of NHL in the post-ART era [7,12,18,26].

### HPV Infection

A broad range of cancers, mostly affecting the anogenital and head and neck areas, are known to be caused by infection with one or more of the high-risk subtypes of HPV [43]. Female PLHIV in China have a high prevalence of HPV co-infection. In one recent study, prevalence of carcinogenic HPV infection among women with HIV was nearly 40% [44]. ICC is a major health problem in China, causing the deaths of approximately 40,000 women annually. We observed 128 cases of ICC in our cohort, for an ASIRC of 47.6 per 100,000 and SIR of 3.8, consistent with previous studies in which females with HIV were estimated to be roughly 5 times more likely to develop ICC [20,21,23,24]. Zhang et al. found a higher SIR of 68.1, and all 14 cancer cases observed among women in the study were ICC [27]. However, in the Taiwan study, incidence of ICC was 413 per 100,000, with SIR at 14.0 [29].

HPV infection is also common among Chinese men who have sex with men (MSM). A recent study in three Chinese cities found high-risk subtype HPV infection among 51% of participants overall, and among 70% of participants with HIV infection [45]. HPV-associated anal cancer has risen to a steady high level among MSM globally [8]. However, very few cases of anal cancer among PLHIV in Mainland China had previously been reported. Zhang et al. found zero cases of anal cancer [27], Yang et al. found 3 among men [28], and we found only 2 (ASIRC: 0.2 per 100,000, SIR: 2.9). A possible explanation for the low incidence of anal cancer thus far observed among men with HIV infection in China is the relatively low prevalence of HIV among MSM during the study period. In our study, only 7.3% of participants reported acquiring HIV infection via homosexual contact. However, the rapid increase of HIV infection in recent years and a high prevalence of HPV infection among MSM in China predicts a rise in anal cancer cases in the future, which may approximate the incidence rate in the Taiwan cohort (45.3 per 100,000, SIR 18.5) [29].

### HBV and HCV Infection

HBV and HCV infection are associated with hepatocellular carcinoma, which accounts for >90% liver cancers. Liver cancer is known to be more prevalent and have higher incidence in east and southeast Asia, with China alone having 50% of all new cases of liver cancer worldwide in 2012 [30]. Incidence of liver cancer is higher among those with HIV in the US and other European countries [5,7,11,12,17,19,23,24]. In China, HIV/HCV co-infection is very common among former blood product donors and recipients as well as people who inject drugs (PWID) [46], and the prevalence of HBV/HIV and HCV/HIV co-infection, and HBV/HCV/HIV triple infection were 8.7%, 18.2%, and 3.3%, respectively, among PLHIV in China’s NFATP [47]. As expected, a high SIR for liver cancer was found by Zhang et al. (6.0, male and female combined) [27], and 14 liver cancer cases were identified by Yang et al., which accounted for 9% of all malignancies observed [28]. In our study, liver cancer had a very high ASIRC among males, 145.7 per 100,000, similar to the Taiwan study (185 per 100,000) [29], and an SIR, 3.9 in males, 5.2 in females, similar to previous studies [5,7,11,12,17,19,23,24].

Other infections may also impact incidence of cancers in PLHIV in China. For example, *Helicobacter pylori* has been associated with stomach cancer. Abovementioned HPV infection has also been associated with penile cancer, oral cavity cancers, other cancers of the head and neck, and non-melanoma skin cancers, and EBV infection has been linked to nasopharyngeal cancer.[8] Incidence rates of all these malignancies were elevated in our study with the one exception—stomach cancer among male PLHIV was not elevated, which is notable since China bears nearly half of the global burden of non-cardia gastric cancers [48].

### Smoking

Previous studies have found that tobacco smoking, as well as the incidence rate of lung cancer are higher among PLHIV than in the general population [3,4,19]. As anticipated, lung cancer incidence was very high in our study—ASIRC of 226.0 per 100,000 among males and 66.8 per 100,000 among females, and SIR of 4.8 for males and 4.2 for females. The incidence rate of lung cancer in our study was higher than the Taiwan cohort [29], which may be attributed to higher prevalence of tobacco smoking in males in Mainland China (53%) than in Taiwan (32–38%) [49,50].

To the authors’ knowledge the present study was the first ever nationwide investigation of the spectrum of malignancies among PLHIV in China and adds to a literature containing few such studies in middle-income country settings. The very large size and nationwide scope of our cohort were two important strengths of our study, which facilitated detection of cancers with low incidence and perhaps improved the overall accuracy of our incidence estimates.

Nevertheless, our study had several limitations. Firstly, only individuals who had been diagnosed with HIV infection could be included in our analysis, and cancer incidence rate in undiagnosed PLHIV remains unknown. Moreover, we could not evaluate cancer cases among the 10.2% of cases (45,261 of 444,712) that were excluded due to either death or loss to follow-up prior to the study’s start or no record of follow-up during the study period. However, a near 90% inclusion rate suggests that our study population is indeed nationally representative. Secondly, because original pathology findings were not included in CRIMS records, their re-examination for accuracy of cancer diagnosis could not be performed. This resulted in some cancer cases lacking a specific diagnosis (classified in our study as ill-defined/unspecified) and likely others being misdiagnosed, both of which may have introduced some miss-classification bias that could have caused under- or over-estimation of some incidence calculations. An example of this may be found in the surprisingly high numbers of brain and CNS cancers observed in our study population. It is likely that some of these cases were, in fact, primary CNS lymphomas [51]. Similarly, it is likely that some cancer cases in our study population went undiagnosed. This too would have biased our results toward under-estimation of cancer incidences. Thirdly, due to the lack of specificity in nearly half of the lymphoma cases in the cohort, we had to classify all lymphomas together into a single group. This prevented comparison of ADC and NADC incidence within the current study and previous studies.

## Conclusion

The findings from this study provide strong evidence of a substantial cancer burden among adult PLHIV in China between 2008 and 2011. Although further study is clearly needed, these results suggest that China should expect that this burden has grown during the period 2012 to 2018, and will continue to do so, as it pushes to further scale up the NFATP in an attempt to meet the UNAIDS 90-90-90 Targets.[40,41] Meeting these goals will undoubtedly result in an overall larger and older Chinese PLHIV population, which will require proactive and accurate cancer screening as well as specialized treatment and case management.

## References

[1] Castro KG, Ward JW, Slutsker L, Buehler JW, Jaffe HW, Berkelman RL. 1993 revised classification system for HIV infection and expanded surveillance case definition for AIDS among adolescents and adults. MMWR Recomm Rep. 1992; 41: 1–19. 1361652

[2] Borges ÁH, Dubrow R, Silverberg MJ. Factors contributing to risk for cancer among HIV-infected individuals, and evidence that earlier cART will alter this risk. Curr Opin HIV AIDS. 2014; 9: 34–40. 10.1097/COH.0000000000000025 24225382 PMC3992916

[3] Rubinstein PG, Aboulafia DM, Zloza A. Malignancies in HIV/AIDS: from epidemiology to therapeutic challenges. AIDS. 2014; 28: 453–465. 10.1097/QAD.0000000000000071 24401642 PMC4501859

[4] Cobucci RNO, Lima PH, de Souza PC, Costa VV, Cornetta Mda C, Fernandes JV et al Assessing the impact of HAART on the incidence of defining and non-defining AIDS cancers among patients with HIV/AIDS: a systematic review. J Infect Public Health. 2015; 8: 1–10. 10.1016/j.jiph.2014.08.003 25294086

[5] Shiels MS, Pfeiffer RM, Gail MH, Hall HI, Li J, Chaturvedi AK, et al Cancer burden in the HIV-infected population in the United States. J Natl Cancer Inst. 2011; 103: 753–762. 10.1093/jnci/djr076 21483021 PMC3086877

[6] Seaberg EC, Wiley D, Martínez‐Maza O, Chmiel JS, Kingsley L, Tang Y, et al Cancer incidence in the multicenter AIDS cohort study before and during the HAART era: 1984 to 2007. Cancer. 2010; 116: 5507–5516. 10.1002/cncr.25530 20672354 PMC2991510

[7] Serraino D, Piselli P, Busnach G, Burra P, Citterio F, Arbustini E, et al Risk of cancer following immunosuppression in organ transplant recipients and in HIV-positive individuals in southern Europe. Eur J Cancer. 2007; 43: 2117–2123. 10.1016/j.ejca.2007.07.015 17764927

[8] Pierangeli A, Antonelli G, Gentile G. Immunodeficiency-associated viral oncogenesis. Clin Microbiol Infect. 2015; 21: 975–983. 10.1016/j.cmi.2015.07.009 26197213

[9] Mitsuyasu RT. Non-AIDS-defining cancers. Top Antivir Med. 2014; 22: 660–665. 25101532 PMC6148886

[10] Clifford GM, Polesel J, Rickenbach M, Dal Maso L, Keiser O, Kofler A, et al Cancer risk in the Swiss HIV Cohort Study: associations with immunodeficiency, smoking, and highly active antiretroviral therapy. J Natl Cancer Inst. 2005; 97: 425–432. 10.1093/jnci/dji072 15770006

[11] Newnham A, Harris J, Evans HS, Evans BG, Møller H. The risk of cancer in HIV-infected people in southeast England: a cohort study. Br J Cancer. 2005; 92: 194–200. 10.1038/sj.bjc.6602273 15583689 PMC2361741

[12] Franceschi S, Lise M, Clifford GM, Rickenbach M, Levi F, Maspoli M, et al Changing patterns of cancer incidence in the early- and late-HAART periods: the Swiss HIV Cohort Study. Br J Cancer. 2010; 103: 416–422. 10.1038/sj.bjc.6605756 20588274 PMC2920013

[13] Mbulaiteye SM, Biggar RJ, Goedert JJ, Engels EA. Immune deficiency and risk for malignancy among persons with AIDS. J Acquir Immune Defic Syndr. 2003; 32: 527–533. 12679705 10.1097/00126334-200304150-00010

[14] Dal Maso L, Franceschi S, Polesel J, Braga C, Piselli P, et al Risk of cancer in persons with AIDS in Italy, 1985–1998. Br J Cancer. 2003; 89: 94–100. 10.1038/sj.bjc.6601017 12838307 PMC2394201

[15] Grulich AE, Li Y, McDonald A, Correll PK, Law MG, Kaldor JM. Rates of non-AIDS-defining cancers in people with HIV infection before and after AIDS diagnosis. AIDS. 2002; 16: 1155–1161. 10.1097/00002030-200205240-00009 12004274

[16] Frisch M, Biggar RJ, Engels EA, Goedert JJ, AIDS-Cancer Match Registry Study Group. Association of cancer with AIDS-related immunosuppression in adults. JAMA. 2001; 285: 1736–1745. 10.1001/jama.285.13.1736 11277828

[17] Serraino D, Boschini A, Carrieri P, Pradier C, Dorrucci M, Dal Maso L, et al Cancer risk among men with, or at risk of, HIV infection in southern Europe. AIDS. 2000; 14: 553–559. 10.1097/00002030-200003310-00011 10780718

[18] Patel P, Hanson DL, Sullivan PS, Novak RM, Moorman AC, Tong TC, et al Incidence of types of cancer among HIV-infected persons compared with the general population in the United States, 1992–2003. Ann Intern Med. 2008; 148: 728–736. 10.7326/0003-4819-148-10-200805200-00005 18490686

[19] Engels EA, Brock MV, Chen J, Hooker CM, Gillison M, Moore RD. Elevated incidence of lung cancer among HIV-infected individuals. J Clin Oncol. 2006; 24: 1383–1388. 10.1200/JCO.2005.03.4413 16549832

[20] Goedert JJ, Coté TR, Virgo P, Scoppa SM, Kingma DW, Gail MH, et al Spectrum of AIDS-associated malignant disorders. Lancet. 1998; 351: 1833–1839. 10.1016/s0140-6736(97)09028-4 9652666

[21] Dal Maso L, Polesel J, Serraino D, Lise M, Piselli P, Falcini F, et al Pattern of cancer risk in persons with AIDS in Italy in the HAART era. Br J Cancer. 2009; 100: 840–847. 10.1038/sj.bjc.6604923 19223894 PMC2653754

[22] Bedimo RJ, McGinnis KA, Dunlap M, Rodriguez-Barradas MC, Justice AC. Incidence of non-AIDS-defining malignancies in HIV-infected versus non-infected patients in the HAART era: impact of immunosuppression. J Acquir Immune Defic Syndr. 2009; 52: 203–208. 10.1097/QAI.0b013e3181b033ab 19617846 PMC2814969

[23] Engels EA, Biggar RJ, Hall HI, Cross H, Crutchfield A, Finch JL, et al Cancer risk in people infected with human immunodeficiency virus in the United States. Int J Cancer. 2008; 123: 187–194. 10.1002/ijc.23487 18435450

[24] Engels EA, Pfeiffer RM, Goedert JJ, Virgo P, McNeel TS, Scoppa SM, et al Trends in cancer risk among people with AIDS in the United States 1980–2002. AIDS. 2006; 20: 1645–1654. 10.1097/01.aids.0000238411.75324.59 16868446

[25] Hessol NA, Seaberg EC, Preston-Martin S, Massad LS, Sacks HS, Silver S, et al Cancer risk among participants in the women’s interagency HIV study. J Acquir Immune Defic Syndr. 2004; 36: 978–985. 15220706 10.1097/00126334-200408010-00013

[26] Simard EP, Pfeiffer RM, Engels EA. Spectrum of cancer risk late after AIDS onset in the United States. Arch Intern Med. 2010; 170: 1337–1345. 10.1001/archinternmed.2010.253 20696958 PMC2921231

[27] Zhang YX, Gui XE, Zhong YH, Rong YP, Yan YJ. Cancer in cohort of HIV-infected population: prevalence and clinical characteristics. J Cancer Res Clin Oncol. 2011; 137: 609–614. 10.1007/s00432-010-0911-y 20532560 PMC11827777

[28] Yang J, Su S, Zhao H, Wang D, Wang J, Zhang F, et al Prevalence and mortality of cancer among HIV-infected inpatients in Beijing, China. BMC Infect Dis. 2016; 16: 82 10.1186/s12879-016-1416-3 26883427 PMC4756453

[29] Chen M, Jen I, Chen YH, Lin MW, Bhatia K, et al Cancer incidence in a nationwide HIV/AIDS patient cohort in Taiwan in 1998–2009. J Acquir Immune Defic Syndr. 2014; 65: 463–472. 10.1097/QAI.0000000000000065 24583616 PMC3995978

[30] Ferlay J, Soerjomataram I, Ervik M, Dikshit R, Eser S, Mathers C, et al (editors). GLOBOCAN 2012: Estimated Cancer Incidence, Mortality and Prevalence Worldwide in 2012 v1.0: IARC CancerBase No. 11 [cited 2019 Mar 18]. Database: International Agency for Research on Cancer [Internet]. Available from: http://gco.iarc.fr/.

[31] Mao Y, Wu Z, Poundstone K, Wang C, Qin Q, Ma Y, et al Development of a unified web-based national HIV/AIDS information system in China. Int J Epidemiol. 2010; 39: ii79–ii89. 10.1093/ije/dyq213 21113041 PMC2992618

[32] Fritz A, Percy C, Jack A, Shanmugaratnam K, Sobin L, Parkin DM, et al (editors). International classification of diseases for oncology. 3rd ed Geneva: World Health Organization; 2000.

[33] National Bureau of Statistics of China. 2010 population census [cited 2019 Mar 18]. Database: National Bureau of Statistics of China [Internet]. Available from: http://www.stats.gov.cn/english/statisticaldata/censusdata/.

[34] Ahmad OB, Boschi-Pinto C, Lopez AD, Murray CJL, Lozano R, Inoue M. Age standardization rates: a new WHO standard. Geneva: World Health Organization; 2001 [cited 2019 Mar 18]. Available from: http://www.who.int/healthinfo/paper31.pdf.

[35] National Cancer Center/Disease Prevention and Control Bureau, Ministry of Health of the People’s Republic of China. Chinese cancer registry annual report: cancer incidence and mortality in Chinese cancer registration areas in 2008. Beijing: Military Medical Science Press; 2011.

[36] Ministry of Health of the People’s Republic of China. 2012 China AIDS Response Progress Report [cited 2019 Mar 18]. Available from: http://www.unaids.org/sites/default/files/country/documents/file,68497,ru..pdf.

[37] Semeere A, Wenger M, Busakhala N, Buziba N, Bwana M, Muyindike W, et al A prospective ascertainment of cancer incidence in sub‐Saharan Africa: the case of Kaposi sarcoma. Cancer Med. 2016; 5: 914–928. 10.1002/cam4.618 26823008 PMC4864821

[38] Zhang T, Shao X, Chen Y, Zhang T, Minhas V, Wood C, et al Human herpesvirus 8 seroprevalence, China. Emerg Infect Dis. 2012; 18: 150–152. 10.3201/eid1801.102070 22257662 PMC3310085

[39] Dukers NH, Rezza G. Human herpesvirus 8 epidemiology: what we do and do not know. AIDS. 2003; 17: 1717–1730. 10.1097/01.aids.0000076337.42412.86 12891058

[40] Joint United Nations Programme on HIV/AIDS. 90-90-90: an ambitious treatment target to help end the AIDS epidemic [cited 2019 Mar 18]. Available from: http://www.unaids.org/sites/default/files/media_asset/90-90-90_en.pdf.

[41] Ma Y, Dou Z, Guo W, Mao Y, Zhang F, McGoogan JM, et al The HIV care continuum in China: 1985–2015. Clin Infect Dis. 2018; 66: 833–839. 10.1093/cid/cix911 29216405

[42] Xiong G, Zhang B, Huang MY, Zhou H, Chen LZ, Feng QS, et al Epstein-Barr virus (EBV) infection in Chinese children: a retrospective study of age-specific prevalence. PloS One. 2014; 9: e99857 10.1371/journal.pone.0099857 24914816 PMC4051769

[43] Muñoz N, Castellsagué X, de González AB, Gissmann L. Chapter 1: HPV in the etiology of human cancer. Vaccine. 2006; 24: S3/1–10. 10.1016/j.vaccine.2006.05.115 16949995

[44] Zhang HY, Fei MD, Jiang Y, Fei QY, Qian H, Xu L, et al The diversity of human papillomavirus infection among human immunodeficiency virus-infected women in Yunnan, China. Virol J. 2014; 11: 202 10.1186/s12985-014-0202-3 25481842 PMC4279793

[45] Li X, Li M, Yang Y, Zhong X, Feng B, Xin H, et al Anal HPV/HIV co-infection among men who have sex with men: a cross-sectional survey from three cities in China. Sci Rep. 2016; 6: 21368 10.1038/srep21368 26892938 PMC4759533

[46] Bao YP, Liu ZM. Systematic review of HIV and HCV infection among drug users in China. Int J STD AIDS. 2009; 20: 399–405. 10.1258/ijsa.2008.008362 19451325

[47] Zhang F, Zhu H, Wu Y, Dou Z, Zhang Y, Kleinman N, et al HIV, hepatitis B virus, and hepatitis C virus co-infection in patients in the China National Free Antiretroviral Treatment Program, 2010–12: a retrospective observational cohort study. Lancet Infect Dis. 2014; 14: 1065–1072. 10.1016/S1473-3099(14)70946-6 25303841 PMC6051428

[48] Casamayor M, Morlock R, Maeda H, Ajani J. Targeted literature review of the global burden of gastric cancer. Ecancermedicalscience. 2018; 12: 833 10.3332/ecancer.2018.833 30679950 PMC6345079

[49] Li Q, Hsia J, Yang G. Prevalence of smoking in China in 2010. N Engl J Med. 2011; 364: 2469–2470. 10.1056/NEJMc1102459 21696322

[50] Adult Smoking Behavior Surveillance System, Health Promotion Administration, Ministry of Health and Welfare. Available at: http://tobacco.hpa.gov.tw/Show.aspx?MenuId=581. Accessed 25 September 2017.

[51] Cesarman E. Pathology of lymphoma in HIV. Curr Opin Oncol, 2013; 25: 487–494. 10.1097/01.cco.0000432525.70099.a4 23942293 PMC4126602

